# Ethnopharmacological uses of *Erythrina senegalensis*: a comparison of three areas in Mali, and a link between traditional knowledge and modern biological science

**DOI:** 10.1186/1746-4269-4-6

**Published:** 2008-03-05

**Authors:** Adiaratou Togola, Ingvild Austarheim, Annette Theïs, Drissa Diallo, Berit Smestad Paulsen

**Affiliations:** 1 Section of Pharmacognosy, Department of Pharmaceutical Chemistry, University of Oslo PO Box 1068 Blindern, 0316, Norway; 2 Département de Médecine Traditionnelle, Institut National de Recherche en Santé Public BP 1746, Bamako, Mali

## Abstract

This paper describes ethnopharmacological knowledge on the uses of *Erythrina senegalensis *DC (Fabaceae) in traditional medicine in three different areas (Dioila, Kolokani and Koutiala) in Mali. Data were collected using interviews of traditional healers selected randomly. The main reported diseases for which *E. senegalensis *was used by the traditional healers were amenorrhea, malaria, jaundice, infections, abortion, wound, and body pain (chest pain, back pain, abdominal pain etc). The fidelity level (which estimates the agreement of traditional healers on the same area about a reported use of the plant) was calculated to compare the results from the three areas. Certain differences were noticed, the most striking was the fact that amenorrhea was the most reported disease in Dioila and Kolokani with 21% of agreement for both areas, while this use was not reported in Koutiala at all. Similarities existed between the three areas on the use of the plant against malaria and infections, although with different degree of agreement among the healers. We also report the results of a literature survey on compounds isolated from the plant and their biological activities. A comparison of these results with the ethnopharmacological information from Mali and other countries showed that some of the traditional indications in Mali are scientifically supported by the literature. For instance, the use of *E. senegalensis *against infectious diseases (bilharzias, schistosomiasis, pneumonia etc.) is sustained by several antibacterial and antifungal compounds isolated from different parts of the plant. The comparison also showed that pharmacologists have not fully investigated all the possible bioactivities that healers ascribe to this plant.

## Background

*Erythrina senegalensis *DC (Fabaceae), locally known as "Nté", is a plant that is used in traditional medicine to cure several diseases. In 2005, a survey was performed in three different regions in Mali (West Africa) to collect information on the uses of seven plants species used in traditional medicine; *E. senegalensis *was included in that study [1]. Little ethnopharmacological information was collected about this plant then since the traditional uses were investigated only in one of the regions where the surveys were performed. As part of the continuing research and development of Improved Traditional Medicine (ITM), ethnopharmacological surveys are being performed by the Department of Traditional Medicine of Mali (the official institute in charge of Malian traditional medicine). Information is collected on the traditional uses of plants that are chosen as source of possible phytoremedies.

The aim of this study is to provide thorough information on the use of *E. senegalsensis *in the traditional practice of three regions of Mali and make a link between this knowledge and information available in the scientific literature on bioactive compounds present in the plant. Based on these results, discussion will be raised whether the healing potential of this tree should be limited to the traditional practice in Mali, or the local knowledge suggests other pharmacological potentials to be investigated in modern bioscience. This may generate new research hypothesis for future studies of this plant. The results of the survey will also add knowledge about the use of *E. senegalensis *from areas in Mali that have not been investigated previously, and will thus be an important background for the possible development of an ITM from the plant.

## Methods

The survey on the ethnopharmacological uses of *Erythrina senegalsensis *was carried out according to the method described in [1]. The healers to be interviewed were selected randomly and no appointment was made prior to the visits. Healers that consented were asked to give their knowledge about the diseases against which they use *E. senegalensis*. Questions were asked about the method of preparation of the remedies, details of administration, including the approximate amounts and number of doses per day or week. The healers were also asked if the remedy had any adverse effect. The interview team was composed of pharmacists, pharmacy students, botanists, and a medical doctor or nurse. The conversations were performed in the national language "bambara" which was fluently spoken by both traditional healers and interviewers, and the information was directly translated and written in English. The conversations were built on trust with the common goal to improve the health situation in the country and to preserve and increase the knowledge on medicinal plants through published data issued from the survey results. This study follows ethical aspects set by the ethical committee of the Malian government. Traditional healers are considered as part of the Malian health care system and a collaboration has been developed by the Department of Traditional Medicine with associations of traditional healers; details of this collaboration and information on the organization of Malian traditional medicine are given in a previous publication [2].

The fidelity level (Fl) among the healers from the same district was calculated according to the following formula:

Fl (%) = (Np/N) × 100

Np is the number of healers from one given district that claim a use of a plant species to treat a particular disease, and N is the number of healers from the same district that use the plants as a medicine to treat any given disease. The formula was applied in order to compare data from different districts where the survey was performed [3]

The literature review was performed within the databases available via the library of the University of Oslo (Norway) (Scifinder, BIBYS, Biological abstract/WebSPIRS and OVID web). The search words were *Erythrina senegalensis *coupled with biological activity.

## Results

### Ethnopharmacological information

The traditional medical uses of *E. senegalensis *in Dioila, Kolokani and Koutiala are presented in Table 1, 2 and 3 respectively. Apart from the commonly known name Nté, traditional healers reported other local names of the plant that were different between the regions. In Kolokani the plant was only called Nté, but in Dioila a variety of local names was reported. These were N'tièmè, Surodolé, and Mousonidé. In Koutiala the plant was called N'gumè, Donitulu and Zoroblé. Several diseases and symptoms were reported by traditional healers against which *E. senegalensis *was used. The main reported ones were malaria, jaundice, infections, gastrointestinal disorders (gastric ulcer, diarrhoea, constipation) amenorrhoea, dysmenorrhoea, sterility, onchocerchosis, body pain (chest pain, back pain, abdominal pain headache and body weakness). The plant was also reported to have wound healing and contraceptive properties (Table 1, 2 and 3).

**Table 1:** Traditional uses of *Erythrina senegalensis *in Dioila

Indications (Number of citation	Plants parts used	Mode of preparation	Added substances	Mode of Administration
Amenorrhoea (22 citation)	Root	powder	*Cola nitida *nut (alternatively)	fumigation of sexual organs,
		maceration		consume in porridge or coffee
		decoction	honey, *Pennisetum sp*	oral
			meat soup or cereal	consume
	Flower	decoction	*Cola nitida *nut	oral, body bath
		powder		consume in porridge
	Bark	powder	*Cola nitida *nut	oral
		infusion		oral
Malaria, Neuralgic malaria (12 citations)	Root	powder		consume in porridge or water
		maceration	roots of *Entada Africana *and *Cochlospermum planchoni*, lemon	oral
		decoction		oral, body bath
	Stem bark	powder		Fumigation, infusion, body bath
		decoction		oral
		maceration		oral
	Leaves and Root	decoction		oral
Jaundice (10 citation)	Root	decoction		oral
		maceration		oral
		powder	water	oral
		infusion		oral
	Bark	decoction		oral
		maceration	*Pennisetum sp*	oral
	Leaves and bark	decoction		oral, body bath
Tonic (9 citations)	Root	decoction		oral
	Bark	decoction	*Pennisetum sp*	oral, consume with porridge
	leaves	decoction		body bath
Gastrointestinal disorders (8 citations)	Root	decoction	*Pennisetum sp*, honey	consume
		maceration	milk	oral
	Leaves	decoction		oral
		powder	porridge	consume
	Bark	powder	porridge	consume
		maceration		oral
Dysmenorrhoea (5 citations)	Root	maceration	*Pennisetum sp*	oral
		powder	water	oral, fumigation
		decoction		oral
	Bark	decoction		body bath
	Flower	powder		consume
Oedema (5 citations)	Root	decoction	root of *Entada africana*, honey	oral, external application on the oedema site
		powder	water	
		macerate	*Pennisetum sp*	oral
Infections (4 citations)	Root	decoction	*Pennisetum sp*	oral, external application
		maceration	*Pennisetum sp*	oral
	Bark	decoction		oral
		maceration	*Pennisetum sp*	oral
Fever (4 citations)	Root	powder	water	oral
	Leaves/root/bark	decoction		oral
Anuria (2 citations)	Root	maceration		oral
	Bark	maceration		oral
Inflammation (2 citations)	Bark	powder	*Butyrospermun parkii *nut butter	external application
		carbonise	banana peal, butter	external application
Contraceptive (2 citations)	Seeds			oral
Somnolence	Root	decoction		oral
Prostate	Root	decoction	spicy rice or millet food	consume
Onchocercosis	Bark	decoction	leaves of *Leptadenia hastata*	oral, body bath
Backache	Bark	powder	*Butyrospermun parkii *nut butter	back massage with ointment
Abortion	Root	powder	fruit of *Aframomun melegueta*, salt	oral
Child night diseases (fever, insomnia)	Root	maceration		oral, body bath
Snake-bite	Root	decoction		external application
Dizziness	Leaves	decoction		steam bath, body bath
Secondary sterility	Leaves	decoction		oral
Nose bleeding	Root	carbonise		nose application
Pneumonia	Root	decoction		steam bat, body bath
Anxiety	Bark	powder		fumigation
Child internal wound	Bark	powder	porridge	consume
Metroragi	Leaves/bark	decoction		oral, body bath

**Table 2:** Traditional uses of *E. senegalensis *in Kolokani

Indications (Number of citation)	Plants parts used	Mode of preparation	Added substances or other plant parts	Mode of Administration
Amenorrhea (4 citations)	Flower	powder	porridge	consume
		macerate		oral
	Bark	decoction	*Pennisetum sp*	oral
	Seeds			oral
Malaria/Icterus (3 citations)	Bark/Leaves	decoction		oral
Chest pain (2 citations)	Bark	Decoction		steam bath
		powder	water	oral
Abortion (2 citations)	Root/leaves/Bark	decoction		oral
Old wounds (2 citations)	Root	Decoction		cleanser
		powder		application to the wound
Gastric ulcer	Root	Powder	Roots of *Boscia angustifolia*, water	oral
Weakness	Leaves + Bark	decoction		oral, body bath
Child abdominal pain	Bark	decoction		oral
Dysmenorrhoea	Root	maceration	Honey (alternatively)	oral
Schistosomiasis	Bark	powder		oral
Diarrhoea	Leaves	decoction		oral

**Table 3:** Traditional uses of *E. senegalensis *in Koutiala

Indications (Number of citation	Plants parts used	Mode of preparation	Added substances or other plant parts	Mode of Administration
Malaria (4 citations)	Bark/root/leaves	decoction/maceration		oral
Jaundice (4 citations)	Bark	maceration	roots of *Cochlospermum planchoni*	oral
		powder/decoction	coffee, thee	oral
	Root	maceration		oral
	Leaves	decoction		oral
Pain: headache/backache (4 citations)	Leaves	decoction		steam bath, back massage fumigation
	Bark	powder	butter	
Infections (3 citations)	Bark	powder	fish soup	consume
	root	maceration	*Pennisetum sp*	oral
	Leaves/root/bark	decoction	*Pennisetum sp*	oral
Gastrointestinal disorders (2 citations)	Bark	powder	porridge	consume
		decoction	porridge	oral
Tonic (2 citations)	Root/leaves/bark	decoction		oral
Nose bleeding	Leaves	decoction		steam bath
Thyphoid fever	Bark	decoction/maceration		oral
Dysmenorrhoea	Leaves/bark	maceration		oral
Internal wound	Leaves/bark	maceration		oral

The Fidelity Level (FL) which estimates the agreement between the traditional healers about a reported use was calculated for the most reported diseases. The results are shown in table 4; the reported uses which did not meet more than 5% of agreement were not included in the table. The FL for the reported diseases was not high in general (21% for the highest FL in this study). This shows a low degree of agreement between the traditional healers on the uses of *E. senegalensis*.

**Table 4:** Comparison of Fidelity level (FL) among traditional healers of the same area on the most reported diseases in Dioila, Kolokani and Koutiala.

**Dioila**		**Kolokani**		**Koutiala**	
		
**Diseases**	**FL**	**Diseases**	**FL**	**Diseases**	**FL**
Amenorrhea	21.3	Amenorrhea	21	Malaria	16.6
Malaria	11.6	Malaria	15.8	Jaundice	16.6
Weakness	8.7	Chest pain	10.5	Backache	16.6
Jaundice	8.7	Abortion	10.5	Infection	12.5
Pain	6.8	Wound healing	10.5	Diarrhoea	8.3
Dysmenorrhea	6.0			Abdominal pain	8.3

The most reported disease against which *E. senegalensis *was used both in Dioila and Kolokani was amenorrhoea. The FL for this use was 21% (the highest FL) for both regions. Interestingly, no report of the plant to cure amenorrhoea was mentioned in Koutiala. This shows a difference between this region and the other two. The use against malaria was reported for all the survey areas with the second highest FL in Dioila and Kolokani (12 and 16% respectively), and the highest agreement in Koutiala (17%). The use of the plant to cure infections reached 12.5% of agreement in Koutiala (third highest FL in this area) and 5% in Dioila (data not included in table 4), while this use was only reported once in Kolokani (table 2).

### Plant parts used and mode of preparation of the remedies

The most reported plant parts used differed between the regions (table 5). In Dioila, more than 50% of the remedies were prepared using the roots, while in Kolokani and Koutiala, the use of the stem bark was predominant (43 and 48% respectively). The leaves, the flowers and the seeds were less used than the root and stem bark in all regions and the two latest were not used at all in Koutiala. The use of the plant material can also depend on its availability. *E. senegalensis *is an evergreen tree, and leaves are always available, but the flowers and seeds have short time of availability. The healers agreed to some extent on the diseases against which this plant was used, but doses varied from healer to healer. The amount of plant part used was "traditionally estimated". The quantity of leaves, trunk bark and root was estimated as handful (250 G approx.). When the plant material was transformed in to powder, the amount to be used was measured as tea spoon, soup spoon, or in fingers (pinch with 2 to 5 fingers gives 10 to 15 mG approx. according to number of finger used).

**Table 5:** Comparison of the plant parts used and the mode of preparation of the remedies between Dioila, Kolokani and Koutiala.

	**Remedies**								
	
**Regions**	Plant parts used* (% of each)					Mode of preparation** (% of each)			
		
	R	SB	L	F	S	D	M	MF	MW
Dioila	53	29	10	6	2	53	21	17	9
Kolokani	22	43	22	9	4	71	12	12	5
Koutiala	30	48	22	-	-	48	38	14	-

* R: Roots; SB: Stem Bark; L: Leaves; F: Flower, S: Seeds.

** D: Decoction; M: Maceration; MF: Mixed with Food; MW: Mixed with Water

The traditional healers from the investigated areas agreed on the non toxicity of *E. senegalensis *remedies. As side effects, diarrhoea and vomiting were reported only once each in Dioila, the first was related to the use of the root powder against malaria, and the second to the use of the same plant part against gastrointestinal disorder. A healer from Kolokani reported that the oral absorption of the stem bark powder for curing chest pain was not to be used during pregnancy.

All the survey areas showed a great similarity on the mode of preparation of the remedies. Decoction was the most reported one (table 5), followed by maceration of the plant parts in plain water or water used for washing the pounded seeds of *Pennisetum sp*. Large quantities of liquids were utilized to prepare the decoctions, especially when the remedy was supposed to be used as a body bath. Most of the remedies were orally administrated and at the same time used to wash the entire body. The amount of remedy to be drunk was also traditionally estimated. It varied from tea glass (approx. 70 mL) to tea cup (approx. 250 mL) for adults and handful of liquid for children. Most of the macerations were left overnight, but in a few cases the maceration took place for 7 days. The time for preparation of decoctions were not precisely given, patients were supposed to proceed the cooking until half of the liquid had evaporated or until the decoction reached a dark colour. In each of the survey area, more than 10% of the remedies were directly mixed with other types of food, e.g. powder preparations were mixed with tea or water (table 5). Other substances (honey, salt, lemon, etc.) were added to the remedies as well (table 1, 2, 3). The healers reported that the taste of the food would mask the taste of the remedies making them easer to be consumed by the patient. In a few cases, plant parts were used directly without any cooking process; they were eaten directly or locally applied on the skin or wounds as a powder (table 1).

### Literature survey of previous studies on compounds with biological activities isolated from *E. senegalensis*

From the literature survey, *E. senegalensis *was found to have been substantially studied and several compounds have been isolated from this plant. Few studies on the biological activities of the compounds isolated directly from the specie *E. senegalensis *were performed, but in the literature it was found that biological activities were tested on the same compounds isolated from other plants or other *Erythrina *species as shown below. The biological activities of the isolated compounds might draw a link between the modern science and the traditional use of the plant.

#### - Antimicrobial, antiviral and antiparasitic compounds

Taylor et al. isolated 2,3 dihydroauriculatin from *E. senegalensis *[4]. The same compound was also isolated from the acetone extract of the root bark of *Ormosia monosperma *Urb. (Fabaceae) that showed moderate activities against oral microbial organisms (*Streptococcus mutans*, *Prophyromonas gingivalis *and *Actinomycess actinomycetemcomitans*) [5].

Erybraedin A is a flavonoid isolated from many *Erythrina *species, like, *E. latissima *E. Mey. (Fabaceae), *E. zeyheri *Harv. (Fabaceae) and *E. senegalensis*. The compound is known as an antimicrobial agent and has been shown to have a strong activity against yeast spores [6]. Erybraedin also showed a high growth inhibitory potency against vancomycin resistant enterococci (VRE) and multiresistant *Staphylococcus aureus *(MRSA). These antibacterial activities were based on bacteriostatic action. The authors also showed that the combination of erybraedin and vancomycin acts either synergistically or additively against VRE and MRSA [7].

The isoflavonoid 6–8-diprenylgenistein isolated from the stem bark of *E. senegalensis *[8] inhibited in vitro 36 different strains of *Staphylococcus aureus *at less than 200 μg/mL, 29 strains of *Shigella spp *and 27 strains of *Salmonellae spp *both between 25 and 200 μg/mL. *Pseudomonas spp *and *Klebsiella spp *were also fairly sensitive toward the compound. All the bacteria strains were clinically isolated from human [9].

Senegalensein, (also named Lonchocarpol A) isolated from the stem bark of *E. senegalensis *[10] exhibited a HIV-inhibitory activity with an IC_50 _of 2.7 μg/mL [11] and an antibacterial activity against *Staphylococcus aureus*, methicillin resistant, and *Enterococcus faecium *vancomycin resistant, with a minimal inhibition concentration value ranging between 0.78–1.56 μg/mL for both bacteria [12].

Alpumisoflavone was isolated from the methanol extract of the stem bark of *E. senegalensis *[8], and also isolated from the chloroform extract of the seeds of *Millettia thonningii *Barker (Leguminosae) and found to prevent the establishment of schistosomiasis infection when applied to mouse skin 2 to 24 hours before exposure to *Schistosoma mansoni*. The mechanism was not understood, but the authors thought that cercariae may be inhibited by the solubilised compounds in the water in which they are swimming [13]. A fraction containing a mixture of alpinumisoflavone and dimethylalpinumisoflavone in a ratio of 23:14 also isolated from the dichloromethane extract of the seeds of *Millettia thonningii*, showed bioactivities against *Schistosoma mansoni *miracidia, cercariae and adult worms. The compounds immobilized miracidia and cercariae, inhibited the egg production and killed all *S. mansoni *adult worms at a concentration of 50 μg/mL after 24 hr of exposure. No miracidia movement was observed after an exposure of only 50 min to the compounds [14].

The presence of these antimicrobial, antiviral and antiparasitic compounds in different parts of *E. senegalensis *might explain and validate its uses in traditional medicine against urinary bilharziosis, gonorrhoea, and various other type of infections in Mali and other areas [1,15].

#### - Cytotoxic compounds

Alpinumisoflavone isolated from *Erythrina indica *Lam. (Fabaceae) was found to be strongly cytotoxic against human solid tumour cells (KB cells). Erysenegalensein E also show a certain degree of toxicity [16]. Wandji et al. have isolated Erysenegalensein E from the methanol extract of the stem bark of *E. senegalensis *[17].

Erythrisenegalone and Senegalensein, two prenylated-flavanones, both isolated from the stem bark of *E. senegalensis *[10,18] have shown anti-tumour promoting activity in vitro in the Epstein-Barr virus early antigens (EBV-EA) inhibition test. The EBV is known to be activated by tumour promotors, including 12-*O*-tetradecanoylphorbol-13-acetate (TPA). Evaluation of EBV-EA inhibition is now used as primary in vitro screening for anti-tumour promoting activity. At the concentration of 1 × 10^3 ^mol, erythrisenegalone and senegalensein were found to show 100% inhibitory activity [19].

Alpinumisoflavone and derrone (the latest also isolated from the stem bark methanol extract of *E. senegalensis*) showed moderate anti-proliferation activity against human leukaemia U937 cells [20].

Cancer diseases are not well known by traditional healers as their practice is symptom directed. Although the same symptoms can be encountered for several diseases, no report of such type of illness was mentioned during the ethnopharmacological surveys in Mali.

#### - Compounds with other activities

Alpinumisoflavone, also isolated from the fruit of *Cudrania tricuspidata *[Bureau ex Lavallée (Moraceae)], significantly inhibited the total mouse brain monoamine oxidase (MAO) activity in a concentration-dependant manner [21]. MAO inhibitors may be a useful therapeutic approach for the treatment of depressive and anxiety disorders, Parkinson's and Alzheimer's diseases [22]. In our field work a traditional healer in Dioila reported the use of the stem bark of *E. senegalensis *to cure anxiety (table 1); although this was reported only once, it might have a link to this tested biological activity.

Erysodine, an alkaloid isolated from the methanol extract of the seeds of *E. senegalensis *[23] was found to be a potent inhibitor of [^3^H]cytosine binding at neuronal nicotinic acetylcholine receptors and a less potent inhibitor of [^125^]α-bungarotoxin binding at muscle-type nicotinic acetylcholine receptors. The potent and competitive nature together with its ability to enter the brain after systemic administration suggest that erysodine may be a useful tool in characterising neuronal nicotinic acetylcholine receptors [24].

Erytrinasinate, an ester isolated from the hexane stem bark extract of *E. senegalensis *[25], showed a weak peroxynitrite scavenging activity [26].

## Discussion

This research contributes to a better understanding of the uses of *E. senegalensis *in traditional practice of Mali. Traditional healers from Dioila, Kolokani and Koutiala reported a variety of local name for *E. senegalensis*. When a plant is known under several local names that are different from one region to another, a sample of plant materiel may be needed during field research to avoid confusion with other plant species. Fortunately in our case, the local name Nté was recognized by the healers all over the different regions.

The healers also reported a variety of ailments against which *E. senegalensis *is used. In some cases, the reported diseases are in fact symptoms only, which indicate that the traditional practice in these areas is symptom directed. This is the case of the traditional practice in various regions of Mali since there are few other means of diagnosis apart from the symptoms reported by the patients [27].

The results of this study show little difference between the regions investigated in the type of the reported diseases apart from the interesting fact the healers from the Koutiala region did not mention amenorrhoea as an important ailment against which *E. senegalensis *was used. This was the most frequent condition mentioned in the other two regions. Jaundice was not mentioned as such in Kolokani.

As reported in the method section, the healers to be interviewed were chosen randomly. Dioila was the region where more healers were interviewed; consequently, more ailments were reported in this region. The potential of a plant to cure a disease can be estimated by its FL [3]. The agreement on the different ailments treated among healers from the same region was low in this study compared to what was observed in our previous study [1] where an agreement up to 54% related to the use of e.g. *Opilia celtidifolia *(Guill. & Perr.) Endl. ex Walp (Opiliaceae) against abdominal pain and 61% against malaria was noticed in the Dioila region. The lower FL observed in the present study can not be attributed to the number of healer interviewed because this number was approximately the same in both studies performed in the same region.

Results of literature research on the traditional uses of *E. senegalensis *in other countries showed some similarities with our studies. *E. senegalensis *was the plant most frequently reported to be used to treat infectious diseases in Guinea [15]. In Siby (Mali) amenorrhoea was the most reported disease against which the plant was used; it is also known to cure urinary bilharzia and eye infections[1]. Doses and treatment durations were not precisely given by traditional healer in Mali. Longuefosse et al. [28] also reported the lack of exact doses in the traditional practice in Martinique. The reason for this may be that the healers did not want the reveal all their knowledge.

The results of the literature research where both chemical compounds and their biological activities were in focus, showed that *E senegalensis *has a great healing potential, especially related to infectious diseases, but also other types of ailments related to the immune system. These results substantiate the use of *E. senegalensis *in traditional medicine in Mali.

However, the healing potential as reported by traditional healers has not been fully investigated by pharmacological studies. Traditional healers strongly supported the use of *E. senegalensis *against amenorrhoea which was the most reported indication in four of the five regions investigated in this and a previous studies [1], no pharmacological evidence was found to support this and several others utilizations which indicates that *E. senegalensis *is a good candidate for biological research. As the flower of *E. senegalensis *is bright red, one might also assume that this use was associated with the so-called signature doctrine, which is a frequent theory of why certain plants are used against a specific ailment. Although some of these reported indications are not well defined or are only symptoms that are encountered in several diseases, other are well defined illnesses (like malaria) that need a pharmacological investigation to substantiate the traditional use. Several of the symptoms or ailments reported could also be covered by a single pharmacological activity. For example, an anti-schistosomal or antifungal activity might be related to traditional indications such as diarrhoea, abdominal pain, and even fever that can be related to bacterial infection. Most of the biological activities reported in the literature are *in vitro *studies, thus further investigations (*in vivo *study) are needed to correlate the ethnopharmacological uses to the biological activity of a particular compound.

Most of the compounds studied have been isolated from organic extracts of the plant material while traditional healers use water extract to cure their patients. The question how these non-water-soluble compounds could be the active principle in phytoremedies normally given as water extracts is interesting. One probable explanation might be the low minimal inhibit concentrations (μg range) of these compounds to give effect. Another could be that co-extraction can take place as plant material often contain compounds like saponins that will enhance the solubility of an otherwise non-soluble compound if present in the same mixture.

## Conclusion

The ethnopharmacological survey showed the healing potential of *E. senegalensis *based on traditional knowledge in the regions of Dioila, Kolokani and Koutiala in Mali. Certain differences about the uses exist between the 3 areas which were estimated by the fidelity level among the traditional healers in the regions. The results of the literature research showed that the antimicrobial activity of this plant has largely been investigated and can explain parts of the traditional uses of *E. senegalensis*. However, when compared with the local knowledge, the literature data are far from giving confirmation of all the activities claimed for this plant. Several traditional uses are still to be explored both *in vitro *and *in vivo *bioassays. *E. senegalensis *is now in our laboratory undergoing further investigations for identification of other chemical compounds having biological activities that may be of importance in the view of its use for production into an Improved Traditional Medicine in Mali.

## Competing interests

The author(s) declare that they have no competing interests.

## Authors' contributions

AT, IA, AT, DD and BSP collected the ethnopharmacological information during the survey. AT and BSP drafted and finalised the manuscript. All authors have read the manuscript and approved it.
